# Evaluation of a Sound Quality Visual Feedback System for Bow Learning Technique in Violin Beginners: An EEG Study

**DOI:** 10.3389/fpsyg.2019.00165

**Published:** 2019-02-12

**Authors:** Angel David Blanco, Rafael Ramirez

**Affiliations:** Music and Machine Learning Lab, Universitat Pompeu Fabra, Barcelona, Spain

**Keywords:** signal processing, audio, violin, learning, e-learning, EEG, music

## Abstract

Current music technologies can assist in the process of learning to play a musical instrument and provide objective measures for evaluating the improvement of music students in concrete music tasks. In this paper, we investigated the effects of a sound quality visual feedback system (SQVFS) in violin learning. In particular, we studied the EEG activity of a group of participants with no previous violin playing experience while they learned to produce a stable sound (regarding pitch, dynamics, and timbre) in order to find motor learning biomarkers in a music task. Eighteen subjects with no prior experience in violin playing were divided into two groups: participants in the first group (experimental group, *N* = 9) practiced with instructional videos and offline feedback from the SQVFS provided in alternation with their performance, while participants in a second group (control group, *N* = 9) practiced with the instructional videos only. A third group of violin experts (players with more than 6 years of experience) performed the same task for comparative purposes (*N* = 7). All participants were asked to perform 20 trials (4 blocks of 5 trials) consisting of a violin bowing exercise while their EEG activity and their produced sound was recorded. Significant sound quality improvements along the session were found in all participants with the exception of participants in the expert group. In addition, participants in the experimental group showed increased interest in the learning process and significant improvement after the second block not present in the control group. A significant correlation between the levels of frontal gamma band power and the sound improvement along the task was found in both the experimental and control group. This result is consistent with the temporal binding model which associates gamma band power with the role of integrating (binding) information processed in distributed cortical areas. Task complexity demands more cognitive resources, more binding and thus, gamma band power enhancement, which may be reduced as the demanded task begins to be automated as it is likely to be the case in both beginners groups.

## 1. Introduction

### 1.1. Feedback in Motor Learning

There is ample literature reporting on the effects of feedback in motor learning tasks. From the first attempts to experimentally test Thorndike's theory of learning (Trowbridge and Cason, 1932), to more recent work (Newell, 1974; Salmoni et al., 1984; Schmidt et al., 1989; Winstein, 1991), studies consistently show how feedback during motor learning increases the rate of improvement over trials. However, the effects on retention and long-term learning are less clear. Approaches providing infrequent feedback have shown improvements in retention phases usually performed 24 h after the experiment (for a review of feedback studies see Winstein, 1991). Still, it is important to consider that this kind of research has focused on studying the effect of feedback in controlled environments where the effect of intrinsic feedback (e.g., visual, auditory, proprioceptive) pertaining to the outcome movement is minimized. This kind of experimental design may imitate the process of learning in a person with sensory deficits who is unable to use intrinsic feedback and depends on the extrinsic feedback (i.e., related to the result of the action) given by the experimenter. On the other hand, motivation is very important in learning (Elwell and Grindley, 1938). Some researchers have attempted to control the motivational effects of feedback in experimental setups where subjects were asked to improve their performance or were given explicit goals (Locke, 1966; Locke and Bryan, 1966) in order to find a significant goal effect. Although those results may relate with the learning of motor skills (e.g., in sports, music), extrinsic feedback could play a different role depending on the task performed, so more specific research is needed in order to understand better the impact of feedback technologies in music students.

### 1.2. Technology-Enhanced Music Learning

Mastering the violin and other bowed-string instruments require special considerations compared with other musical instruments. As opposed to the piano, for instance, pitch control in the violin is continuous and correct intonation is an important issue. In addition, the process of good sound generation in the violin is a notorious complex task which requires precise spatiotemporal control of bowing gestures (Schoonderwaldt and Demoucron, 2009). Acquiring correct bowing motor skills require many hours of practice in which aural feedback is crucial for students to adjust their motor gestures to generate good sound. According to Konczak and Jaeger (2009) novice players need approximately 700 practice hours to achieve bowing skills comparable to those of experts. Moreover, string players have the highest risk of playing-related musculoskeletal injuries/disorders (PRMDs) with the neck and shoulder being the main body parts affected (Middlestadt and Fishbein, 1989).

A recent survey on Australian higher education music students showed how the use of Youtube and self-recording has become common practice among them (Zhukov, 2015). Youtube offers videos of professional musicians performing music repertoire pieces as a model for students while self-recording has become an important tool for self-evaluation. Previous research (Kepner, 1986; Bundy, 1987) found that high school instrumentalists were more able to identify musical errors when hearing tape recordings of their own performances than when actually performing the pieces. In particular, Bundy (1987) explains the obtained results by a sensory blocking theory which hypothesizes that when musicians are concerned with monitoring a big number of sensory aspects involved in performance (like sight-reading or finger movements) the sense of hearing, which is perceived to be of lesser importance, is blocked. However, recent research (Hewitt, 2001) studied the effects of listening to a model (i.e., an expert reference performance), listening to oneself on audiotape, and self-evaluation on junior high school instrumentalists, concluding that there is a significant interaction effect for modeling and self-evaluation. However, self-evaluation (which in the case of the study consisted on the Woodwind Brass Solo Evaluation Form Saunders and Holahan, 1997) or self-recording on their own were not found to be effective strategies for improving music performances. Although self-recording may be important, in absence of a teacher it requires the student to be his/her own judge which may be problematic. The superiority of highly trained musicians encoding spectral and temporal features of music-sound compared with non-musicians has been found in a large number of neuroscientific studies (Besson et al., 1994; Koelsch et al., 1999; Pantev et al., 2001; Tervaniemi et al., 2005; Hutchins and Peretz, 2012). For example, Koelsch et al. (1999) demonstrated, using electrophysiological and behavioral data, that highly trained violin players are able to detect automatically undetectable pitch differences for nonmusicians.

Current music technologies provide us with objective measures of student improvement in specific music tasks. Thus, such technologies can allow us to monitor the learning process of music students in order to provide better and personalized learning strategies. In addition, objective measures about music students' performance may serve as additional information which could complement the verbal feedback given by the teacher. In the past, the role of feedback in music learning has been addressed mainly to study the effects of real-time visual feedback (RTVF) in singing. Welch et al. (1989) studied the effect of a feedback system called SINGAD (Singing Assessment and Development) in 32 primary school children aged 7 years. The system provided a real-time F0 trace plotted against time together with the target notes displayed in order to guide time and pitch accuracy. The study reported improved pitch accuracy by using the system. Previous research has studied the effect of using different kinds of interfaces and different kinds of feedback in singing voice (Thorpe, 2002; Welch et al., 2004; Wilson et al., 2008; Leong and Cheng, 2014), trombone (Schlegel and Gregory Springer, 2018), piano (Hamond, 2017), and violin (Wang et al., 2012). Although there are differences in the way RTVF may improve performance, most of the previous studies reported beneficial effects of RTVF in learning. An extensive review on feedback and technology applied to music learning can be found in Hamond (2017). The same author also investigated the nature and application of combined visual-auditory feedback generated by technology systems in higher education piano learning and teaching contexts. As suggested by self-reports collected from music students, the feedback provided could increase conscious-awareness of their own performance. As related by one of those students: “Sometimes you know in your mind what you want to do, […] but sometimes you do not realize exactly what you're doing in practice[…]. So, when you hear, you can clearly see what you are doing and what you're not” (Hamond, 2017, p. 278).

Regarding violin learning, special efforts have been done to offer different kinds of feedback, not only on the produced pitch but also on timbre, good posture, and bowing technique. The i-Maestro project (Ng and Nesi, 2008) was one of the first steps in that direction offering tools based on gesture analysis and audio processing. More recently the TELMI project has developed tools for providing feedback on timbre quality, pitch and timing accuracy, posture and bowing techniques, and musical expression (Ortega et al., 2017; Dalmazzo et al., 2018; Giraldo et al., 2018; Zacharias et al., 2018). Optical motion capture combined with sensors has also been used to extract bowing parameters from violin performance (Schoonderwaldt and Demoucron, 2009; Deutsch, 2011) allowing to study and compare the motor patterns of professional and student violinists. Tracking violin performance using low-cost methods has also been investigated by Perez Carrillo and Wanderley (2012) through the sole use of audio signal and a system trained on empirical data previously collected with a highly accurate sensing system. Pardue et al. (2015) also explored low-cost methods using a resistive fingerboard and four optical reflectance sensors placed on the bow stick. Some attempts have been done in order to evaluate motion capture techniques to teach violin skills. For example, Van Der Linden et al. (2011) used a wearable system to teach good posture and bowing technique to novice violin students and found a larger improvement when compared with a control group of subjects who received the same number of training sessions using conventional teaching techniques. One possible limitation of the previously mentioned study is that the quality of generated sound is not taken into account, while in violin learning the production of a good sound is one of the main reasons for learning a correct bowing technique.

The work of Romaní et al. (2015) aimed to identify audio descriptors, extracted from the recordings of professional musicians while playing single notes, maximally correlated with their own subjective opinions about the quality of the produced sound. Some of the features that showed higher correlations were those characterizing pitch stability and dynamic stability. This research led to the implementation of Cortosia (Korg, 2018) an app owned by the Korg company, which aims to provide students with visual feedback about the quality of their produced sound. More recently, Giraldo et al. (2018) investigated the application of machine learning techniques to obtain sound quality model and implemented a real-time feedback system for enhancing violin learning. However, no studies have been done until now to evaluate the pedagogical effectiveness of such systems.

One could be tempted to offer simultaneous real-time feedback in violin learning environments (e.g., violin-bow orientation, bowing trajectory, and timbre quality). However, a common concern found in user studies offering several simultaneous feedback is that participants usually have difficulties dealing with them (Van Der Linden et al., 2011; Johnson et al., 2012; Johnson, 2014). Delivering the different feedback separately at different times and as requested by the user could be one possibility to resolve that problem, as has been the approach in the TELMI project. Another common concern is the potential dependency that feedback systems could create on students.

Recent research (Brandmeyer et al., 2011) has evaluated the effects of RTVF on expressive percussion performance interpreting their results using the Cognitive Load Theory (CLT) (Paas et al., 2003). In their work, they differentiate between three different kinds of cognitive load: intrinsic, extraneous and germane. Intrinsic cognitive load is associated with the difficulty of the particular task whereas extraneous cognitive load relates to the manner in which information is received. On the other hand, germane cognitive load relates to the mental resources involved in learning in general, independently of the task. Brandmeyer et al. (2011) found empirically that too many visual elements can create a high extraneous cognitive load in participants, dividing their attention and leading to poorer learning outcomes. However, apart from behavioral measures, no other measures were used to evaluate the amount of cognitive load participants were experiencing. Physiological measures can provide objective measures of the mental work a person is experiencing while learning. Recently, the neural activity associated with learning tasks has been investigated by the neuroscientific community using both functional neuroimaging and electroencephalography (EEG) techniques. In particular, EEG is the most common technique used to study cognitive load from brain activity and one of the most feasible among other electro-physiological measures (Miller, 2001).

### 1.3. E-Learning Systems Inspired in Brain Activity (EEG)

Event-related (de)synchronization (ERS/ERD) is a well-established measure for the quantification of changes in different frequency bands of the EEG signal. It reflects the decrease (desynchronization) or increase (synchronization) in a band power during a test (time period where the subject is performing a specific task which demands cognitive load) compared with a reference baseline (time period without any task demands). This is usually done for each electrode. A positive ERD/ERS value means a decrease in a band power (desynchronization, ERD) while a negative value indicates an increase in band power (ERS). It has been reported repeatedly for several researchers that alpha and theta band activity (8–13 Hz and 4–7 Hz, respectively) is very sensitive to task difficulty or cognitive load in a wide variety of task demands (Klimesch, 1999; Gevins and Smith, 2003; Neubauer et al., 2006). Generally, as cognitive load increases, frontal midline theta band increases, and posterior alpha band decreases. Larger alpha band ERD has been associated with highly intelligent subjects and good performance (Jaušovec and Jaušovec, 2004). Explanations of this phenomenon are usually delegated to the neural efficiency hypothesis which assumes that high alpha band power reflects cortical inhibition. On the other hand, theta has been investigated for its implications in memory performance (Raghavachari et al., 2006) showing strong increases in the frontal area during the encoding and retention period (Maurer et al., 2015). Thus, an alpha band power decrease at posterior sites (larger alpha band ERD) and a frontal theta increase represent a general index for cognitive demands. Some research also highlights the importance of gamma band waves (30–100 Hz) which its enhancement is observed within a task-specific spatial distribution (Fitzgibbon et al., 2004) and seems to be correlated with cognitive load in humans (Howard et al., 2003). The temporal binding model gives gamma band the responsible role of integrating (binding) information processed in distributed cortical areas. Task complexity demands more cognitive resources, more binding and thus, gamma band power enhancement. Interestingly, some research has found that subjects with musical training show enhanced induced gamma band activity (Shahin et al., 2008; Trainor et al., 2009) suggesting it reflects a superior binding of acoustical features (e.g., pitch, timbre, harmony) and processes also thought to be enhanced by music training, e.g., anticipation, expectation and attention (Bhattacharya et al., 2001; Sokolov et al., 2004; Gurtubay et al., 2006).

The viability of the use of EEG to test the effectiveness of learning materials designs has been provided by some studies (Antonenko and Niederhauser, 2010; Antonenko et al., 2010). Thanks to the measure of participants' cognitive load it is possible to assess which learning strategy seems to work better in concrete situations. On the other hand, some studies have also started to investigate the potential of real-time monitoring of mental workload to improve human performance. For instance, Kohlmorgen et al. (2007) describes a system to reduce distractions while driving by monitoring mental workload.

EEG has also been used to improve music performance through the use of an increasingly popular technique called neurofeedback. It consists of learning, through visual or auditory feedback, how to modify voluntarily your own mental activity. Several studies have reported improvements in the music performance of those musicians who received a neurofeedback session on the theta /alpha protocol (i.e., learning how to maximize the theta to alpha ratio) before a performance, compared with other groups who received different kinds of relaxing techniques like the Alexander technique or different neurofeedback protocols (Bazanova et al., 2009; Gruzelier, 2009). Similar results have also been found for dancers (Raymond et al., 2005). According to the authors, the production of theta waves with eyes closed is related to the hypnogogic process which at the same time is associated with an improvement of the creative process and well-being of users.

Other studies have tried the use of theta-EEG and EMG biofeedback with violinists while they perform, with positive results (Silvana et al., 2008). The pre-recorded sound of applauses as feedback gave the musician the opportunity to recognize which is the adequate mental and muscular state needed for optimum performance. The reason to train theta during the performance was that some investigations have found enhanced theta activity in highly-skilled professional musicians (Klimesch et al., 1997; Bazanova and Aftanas, 2006). According to the neural efficiency hypothesis experts should show lower brain activation (which means higher theta power and more efficient networks), and thus, training students to learn how to use their brain more efficiently could lead to an enhancement of their performance.

The relationship between EEG power changes and proficiency have also been reported in sports activities such as rifle marksmanship (Haufler et al., 2000; Kerick et al., 2004), archery (Salazar et al., 1990; Landers et al., 1994) and golf (Crews and Landers, 1993; Babiloni et al., 2008). This research shows how the most predictive data of expertise is recorded before the skilled movements occur, in what is called the “pre-shot routine.” For instance, it has been shown that the magnitude of the increase in theta power before the shot is correlated with the accuracy of the shot. Berka et al. (2010) tracked the learning process of beginners in rifle marksmanship while firing a total of 40 shots and correlated the accuracy of the results with the EEG power activity, finding increases in theta and high theta Bands (6–7 Hz) just as experts showed during all their trials. They also compared the results of the learning group with another one which, additionally, received a neurofeedback training based on the same frequency bands showing how the neurofeedback group obtained significantly better results. Similar results were also found by Gentili et al. (2008) where subjects had to learn and interact with new tools. They found increases in alpha and theta band power in the frontal and temporal lobes during movement planning (i.e., just before the movement, like in the pre-shot routine).

However, in a recent study (Gutierrez and Ramírez-Moreno, 2016) changes in brain activity associated with the progression of the learning experience were estimated with different results. They monitored the process of learning to typewrite using the Colemark keyboard layout, which is an alternative to the QWERTY layout, finding a decreasing trend of the beta and gamma bands. They interpreted beta band decrease as a result of long-duration repetitive hand movements, similar to results found by as Niemann et al. (1991) and Erbil and Ungan (2007), and explained the gamma band decrease as a consequence of the temporal binding model previously mentioned, which associates gamma band activity with coupling perception and learning, as reported by Gruber and Müller (2005).

### 1.4. Aims of the Present Work

The aim of this work is to contribute to the understanding of the effects of feedback in music learning from an electrophysiological point of view. For this purpose, we have evaluated the effectiveness of using a sound quality visual feedback system (SQVFS) to improve the quality of sound produced by of novice violin players while their EEG activity and the violin sound they produced was recorded. These recorded data provides non-invasive biomarkers of motor learning in a musical task. Participants (with no previous experience with violin or any other bowed string instrument) were asked to produce a stable and sustained violin sound on an open string (i.e., the second string in the violin). The choice of using an open string was to allow participants to exclusively concentrate their attention to control the bow movement. This task requires to control and change the pressure of the bow along the whole movement due to the fact that bow pressure requires to be heavier at the frog and lighter at the tip. If the pressure of the bow is not constant along the movement both pitch and energy of the produced tone could change. For that reason, we hypothesized that the use of dynamic stability and pitch stability audio descriptors, as Romaní et al. (2015) did, to measure sound quality among trials would allow us to track improvement through the session. We also offered the numerical result of the descriptors as feedback to the participants (i.e., the SQVFS).

Participants were divided into two groups. Both of them had access to learning materials and reference videos during the experiment, but in addition one of the groups received offline feedback about the quality of their performance given by the SQVFS. The quality of the produced sound, as well as the EEG activity of each participant, was recorded during 4 blocks of 5 trials each (20 trials in total). An additional group of violin experts was considered in the experiment for comparative purposes. Data recollected in this study is publicly available in Zenodo (Casares and Ramírez, 2018) and the code to analyze it in Github (Blanco, 2018).

## 2. Materials and Methods

### 2.1. Participants

The study was carried out in the recording studio located in the Information and Communication Technologies Engineering (ETIC) department of the Universitat Pompeu Fabra, Barcelona and included the participation of twenty-five right-handed subjects. Participants conceded their written consent and procedures were approved by the Conservatoires UK Research Ethics committee on 04/04/2017, following the guidelines of the British Psychological Society. Participants provided information about their musical skills, main instrument and years of music training. Those with extensive experience in violin playing were included in the expert group [EG;6 male, 1 female; mean age: 35.2 (9.01); mean years studying violin: 7.6 (2.19)]. Participants with no violin (or viola, double-bass or cello) experience were included in the beginner's group. This last group, was randomly divided in two groups: the first group [BF; 6 male, 3 female; mean age: 27.57 (4.46); all of them were musicians with several years of experience, mean: 9 (5.07)] practiced with instructional videos and offline feedback from the SQVFS reflecting the quality of their produced sound, while the second group [BNF; 8 male, 1 female; mean age: 27.2 (2.28)] practiced with the instructional videos only. All participants were musicians with several years of experience, mean: 10.8 (4.65).

### 2.2. Materials

**Figure 1:**
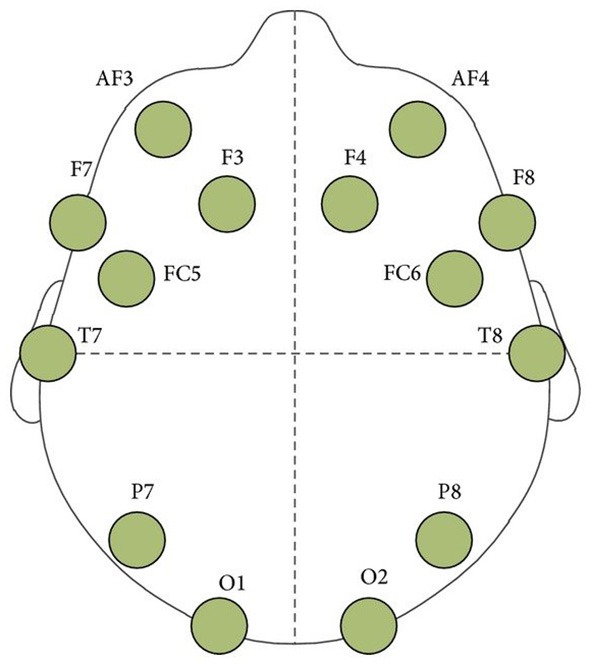
Emotiv EPOC electrodes aligned with positions in the 10–20 system.

EEG data were acquired using the Emotiv EPOC EEG device. The Emotiv EPOC consists of 16 wet saline electrodes, located at the positions AF3, F7, F3, FC5, T7, P7, O1, O2, P8, T8, FC6, F4, F8, AF4 according to the international 10-20 system (see Figure 1). The two remaining electrodes located at P3 and P4 are used as reference. The data acquired were digitized using the embedded 16-bit ADC with 128 Hz sampling frequency per channel and sent to the computer via Bluetooth. The Emotiv Control Panel software was used to monitor visually the impedance of the electrodes contact to the scalp. The data were recorded using the OpenViBE platform (Renard et al., 2010) and later processed in EEGLAB (Delorme and Makeig, 2004) under the Matlab environment (MATLAB, 2010).

A Zoom H4N handy recorder was used to record the audio of each trial which was processed in Matlab using the “Yin pitch estimation toolbox” (Llimona, 2015) in order to extract audio features for assessing sound quality and provide feedback to participants. Yin is a widely used algorithm to estimate fundamental frequency both in speech and music (De Cheveigne and Kawahara, 2002).

**Figure 2:**
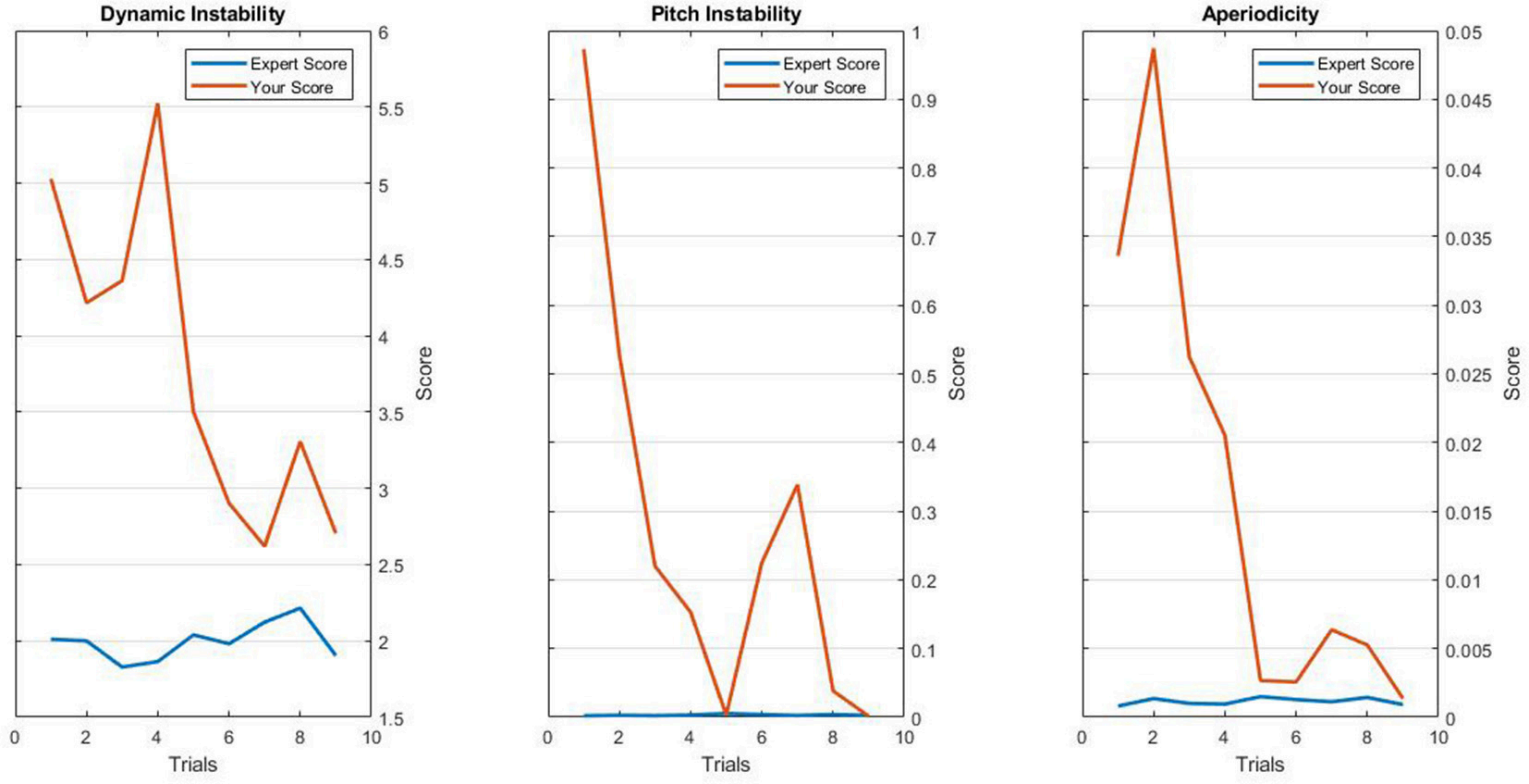
Example of the results in dynamic instability, pitch instability and aperiodicity shown to a subject just after performing trial number nine.

Visual feedback provided to the BF group consisted of graphs generated in Matlab showing the sound quality score in the y-axis and the trial number in the x-axis. Feedback was intended to allow participants to monitor their progress and compare their performance to that of an expert participant who previously did the experiment (also plotted in the feedback screen) (see Figure 2).

**Figure 3:**
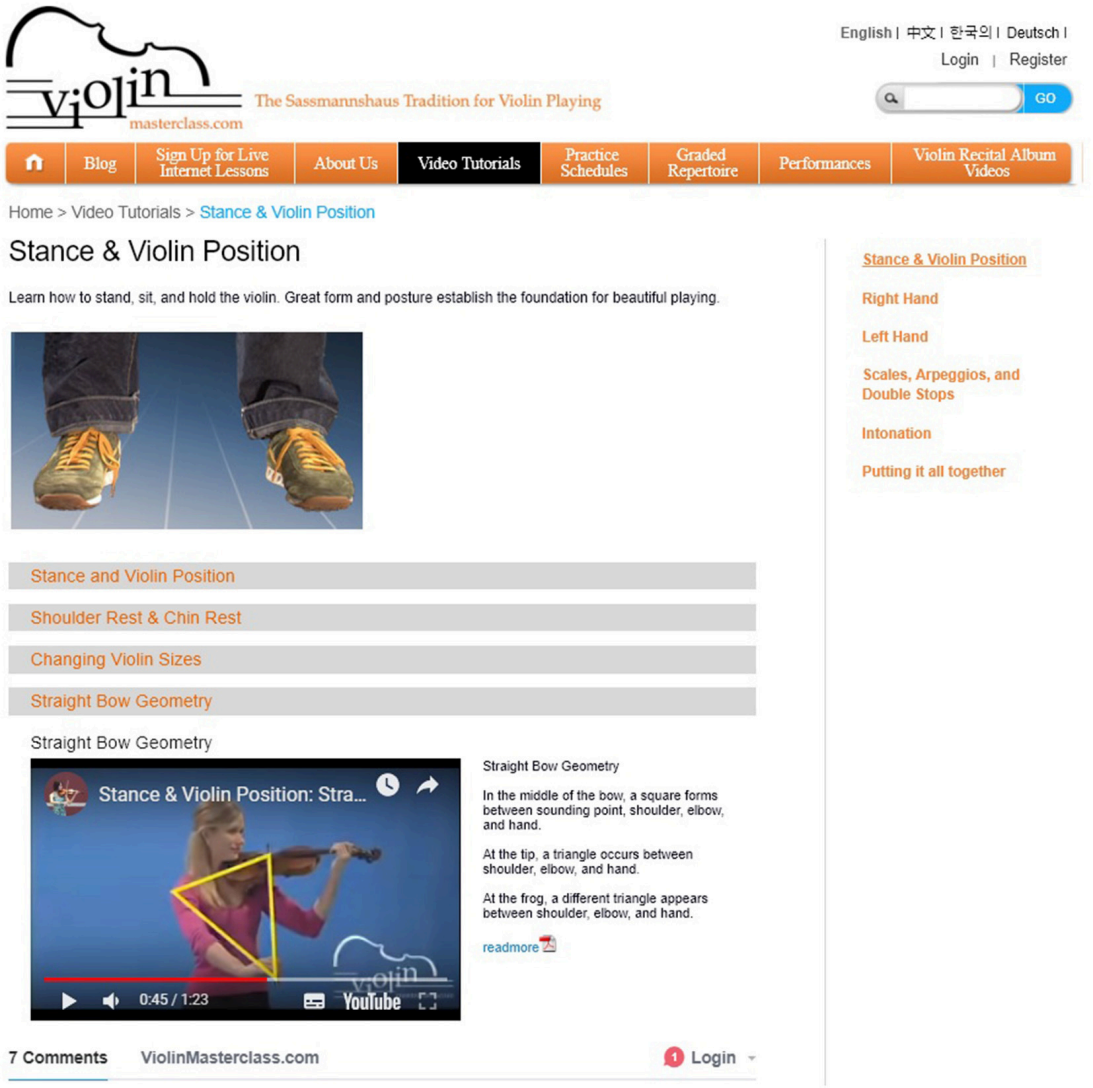
Instructional videos on stance, violin position, straight bow geometry and bow grip were collected from Violinmasterclass.com.

Instructional videos about basic violin playing techniques, e.g., stance, violin position, bow position, and grip, were used to provide participants with basic information. The videos were collected from the web (Sassmannshaus, 2018) (see Figure 3). In addition, we recorded a reference video of the requested task performed by a professional violin player. The produced video was shown to all participants to explain the task to be performed. The video can be found in Zenodo (Casares and Ramírez, 2018).

**Figure 4:**
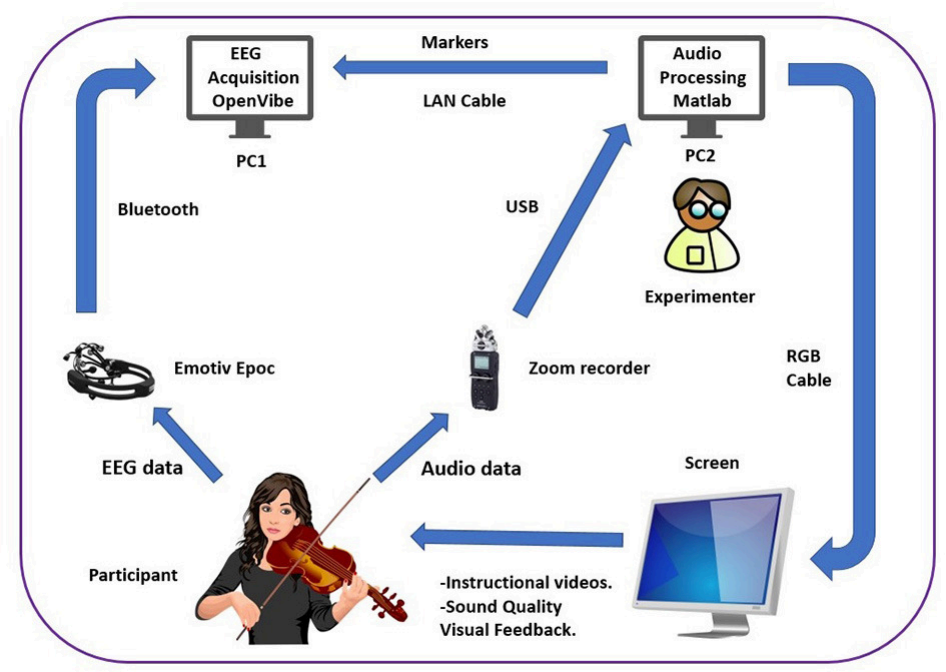
Setup of the experiment. EEG data and audio data from the participant are processed separately in different computers that are communicated through OSC. The experimenter controlled the display of instructional videos (including the reference expert video) for both BNF and BF groups and sound quality visual feedback for the BF group.

EEG acquisition and audio processing were performed on different laptops (PC1 and PC2, respectively). To synchronize audio and EEG data PC2 sent markers to OpenVibe in PC1 through OSC everytime a new trial began and ended. The experimenter controlled the display of instructional videos and the reference expert video for both BNF and BF groups and sound quality visual feedback for the BF group (see Figure 4).

### 2.3. Methods

Due to the nature of the experiment, it was not possible to conduct a double-blind study. In order to avoid unconscious bias during the instructions given to participants, both beginner groups (i.e., BNF and BF) watched the same set of instructional videos on violin and bow position and stance with a total duration of 10 min (Sassmannshaus, 2018). Participants watch the videos while the EEG device was positioned on their heads. Once setup of the EEG device and the videos were finished, participants proceeded to perform the violin bowing exercise which consisted in the alternation of eight up and down bowing movements using the full length of the bow with the goal of producing a sound in the A open string. Participants were asked to produce a stable and sustained sound at the same tempo of the reference video. Participants were also asked to minimize blinking and facial movements during the exercise to avoid artifacts in the EEG signal.

**Figure 5:**
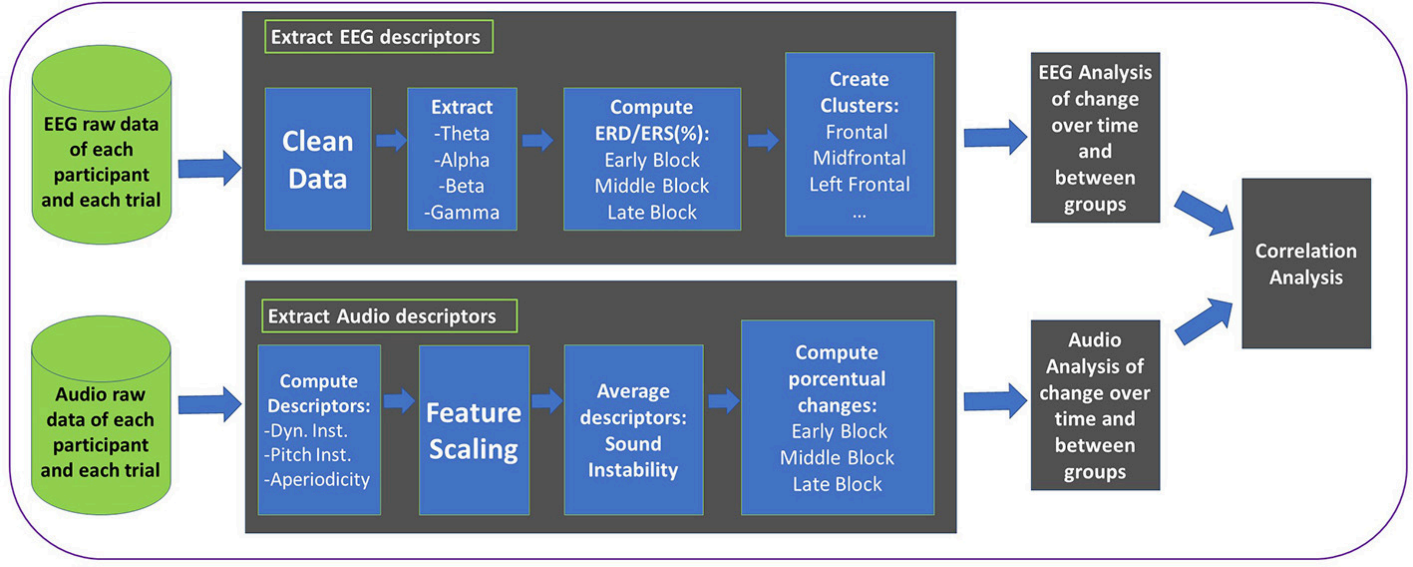
EEG and audio raw data from each participant and each trial were processed separately to extract meaningful descriptors that were used to analyze changes over time and between groups together with correlations.

The blocks of trials were named as follows: early block (trials from 6 to 10), middle block (trials from 11 to 15), and late block (trials from 15 to 20). In Figure 5 we can see all the steps that were involved in the processing of audio and EEG data. First, audio and EEG data were processed separately to extract meaningful descriptors. Posterior EEG and audio analysis allows us to study changes over time and between groups. Finally, the correlation analysis allows us to measure the correlation between sound quality features and EEG features. We also did a behavioral analysis to study participants' learning patterns, i.e., number of times they consulted the learning materials. The total duration of the experiment was approximately 45 min. The first block of trials, where both groups of beginners did not have the option to rewatch instructional videos or offline feedback from the SQVFS, was used as a baseline to compute the amount of change in both sound quality and EEG waves around the rest of blocks. From the early block on, BF and BNF had the option to rewatch both instructional and/or reference expert videos as many times as they wanted for the rest the trials. In addition, the BF group had the opportunity to receive offline feedback from the SQVFS visualizing the dynamic stability, pitch stability and aperiodicity scores of their performance for each trial. The number of times a participant requested the learning materials were also recorded.

#### 2.3.1. Extraction of Audio Features

Violin sounds generated by participants were recorded for each trial with a sampling rate (SR) of 44,100 samples. The Yin algorithm was used to extract sound descriptors from the audio signal of each trial using a windows size of 33 ms and a hop size of 0.7 ms. Three different parameters were computed for each window: instantaneous power, fundamental frequency (f0) in cents (reference: 440) and aperiodicity. The quality of the sound recorded in one trial may be assessed through sound descriptors such as dynamic stability (see 1) or pitch stability (see 2) by computing the standard deviation of both f0 and power throughout the trial (Romaní et al., 2015). Aperiodicity was also included as a descriptor (details about how aperiodic power is computed can be found in De Cheveigne and Kawahara, 2002). See Equations (1–3) for a formal definition of these descriptors.

(1) $$dynamicStability\;=\;\frac{1}{\sqrt{\frac{1}{N}\sum_{i=1}^{N}{\left(p_{i}-\mu \right)}^{2}}}\;$$

(2) $$pitchStability\;=\;\frac{1}{\sqrt{\frac{1}{N}\sum_{i=1}^{N}{\left(f0_{i}-\mu \right)}^{2}}}$$

(3) $$aperiodicity\;=\;\frac{aperiodicPower}{totalPower}$$

First, the values of pitch stability and dynamic stability were inverted and renamed pitch instability and dynamic instability, respectively. After that, the values of aperiodicity were standardized together with pitch instability and dynamic instability by mean subtraction and averaged for each trial. The descriptor resulting from this process was called Sound instability. In order to check the utility of using aperiodicity, we computed Sound instability in two different ways: one including aperiodicity, and the other not including it. Audio features' discriminability between beginner and expert players was investigated by computing the information gain for each feature [the Gain Attribute Evaluation (IGAE) implementation in Weka (Witten et al., 2016) was used to rank the features] over the first five trials (before receiving any kind of external feedback).

#### 2.3.2. EEG Power Computation

For each subject and each single-trial, the power spectral density (PSD) was computed from activity in each electrode using Welch's overlapped segment averaging estimator using a window size of 2 s. Four frequency bands were extracted corresponding to theta (4–8 Hz), alpha (8–13 Hz), beta (13–24 Hz), and gamma (30–50 Hz). Changes in the EEG signal were computed in the form of event-related desynchronization (ERD) or an event-related synchronization (ERS). In the ERD/ERS equation (see 4) the baselineIntervalBandPower corresponds to the PSD computed during the first block of five trials while testIntervalBandPower corresponds to the PSD computed for each other block.

(4) $$ERD/ERS(\%)\;\;=\;\;\frac{baselineIntervalBandPower-testIntervalBandPower}{testIntervalBandPower}*100$$

Outliers were removed for each trial using the modified Z-score equation (see 5) to label as potential outliers those modified Z-scores with an absolute value greater than 3.5 as Iglewicz and Hoaglin (1993) recommend.

(5) $$M_{i}\;=\;\frac{0.6745(x_{i}-\tilde{x})}{median(|x_{i}-\tilde{x}|)}$$

Where $\tilde{x}$ denotes the median, i.e., the denominator is the median absolute deviation (MAD).

Electrodes were grouped and averaged into different clusters: frontal (AF3, F7, F3, FC5, FC6, F4, F8, AF4), midfrontal (F3,F4), left frontal (AF3, F7, F3, FC5), right frontal (FC6, F4, F8, AF4), posterior (P7, O1, O2, P8), left posterior (P7,O1), right posterior (O2,P8) occipital (O1, O2), left parietal (P7), right parietal (P8), temporal (T7,T8), left temporal (T7), right temporal (T8). The different frequency bands for each cluster formed the initial amount of features for each group of participants. Information gain was computed for each feature over the first five trials to find those features that discriminate better between beginners and experts.

## 3. Results

### 3.1. Audio Analysis

**Figure 6:**
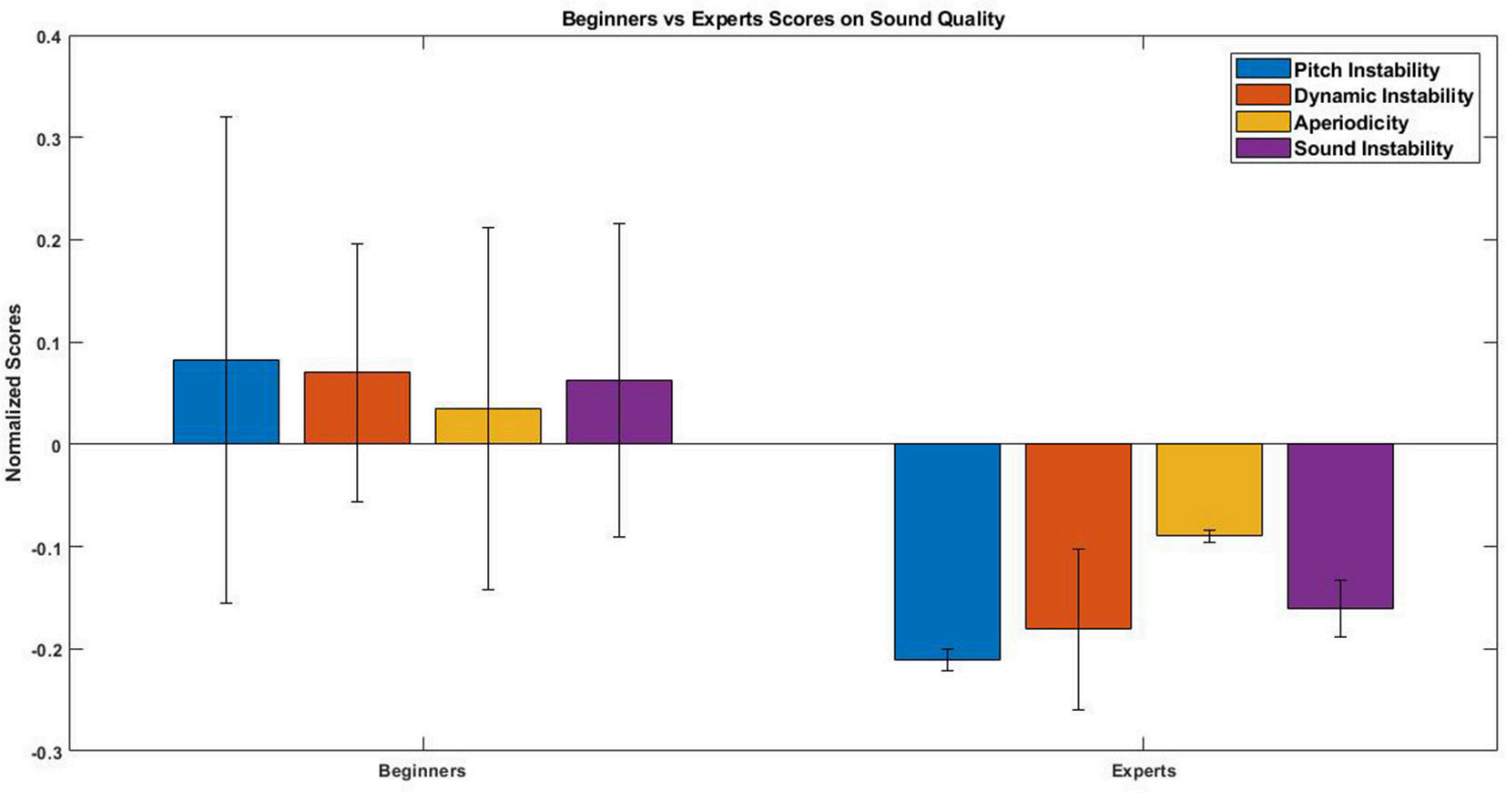
Median and standard deviation of each audio descriptor during the first block (5 trials). Both groups of beginners are represented together. As expected, descriptors show how the sound of beginners is more unstable than the one of experts.

The results of IGAE ranked the Sound instability descriptor which included aperiodicity as the most important one to differentiate between beginners and experts with a value of 0.668. It was followed by pitch instability with 0.599; dynamic instability with 0.577; Sound instability without aperiodicity with 0.549 and aperiodicity with 0.514. A Shapiro-Wilk test for normality was performed on the data showing significant results leading us to use non-parametric statistical tests. A Wilcoxon rank-sum test was performed for each audio descriptor comparing experts and beginners showing significant results (*p* < 0.00001 for all the descriptors). Beginners showed higher values than experts, i.e., beginners produced more unstable sound (see Figure 6).

**Figure 7:**
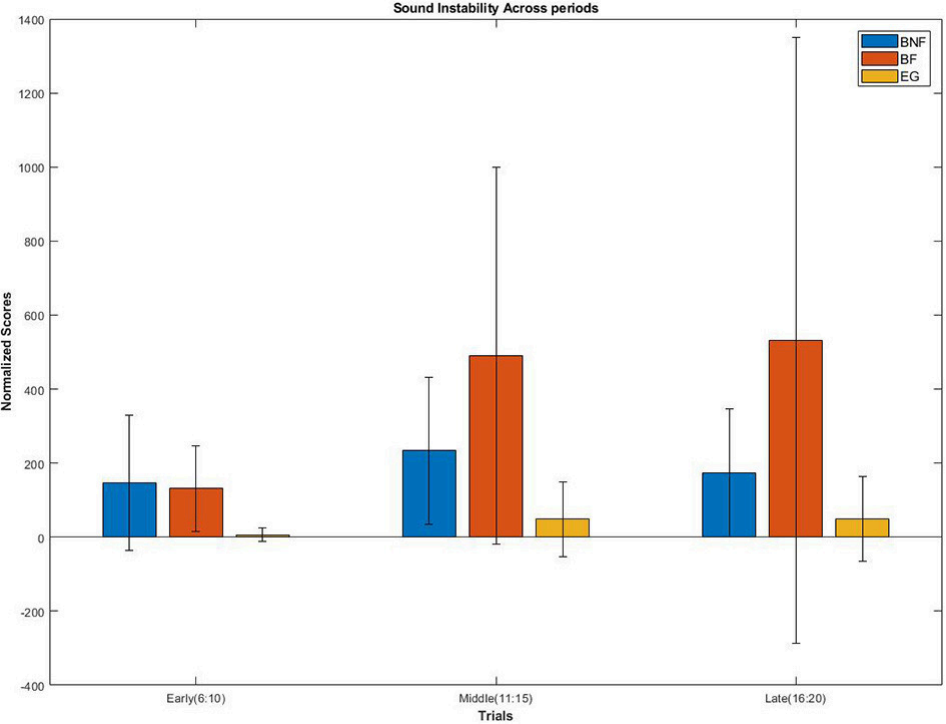
Porcentual changes of each experimental group across blocks on Sound instability scores. Both beginners groups showed significant differences compared with the baseline but only the BF group showed significant differences between blocks.

Percentage changes of Sound instability were computed for each block and for each group using the first block as a baseline. After adjusting the *p*-value for three tests using the Bonferroni method, significant changes through blocks were found for both beginners groups but not for the experts (Wilcoxon sign-rank test, BNF: *p* = 0.00001; BF: *p* = 0.000005; EG: *p* = 0.1396). In order to detect differences between the blocks, three more Friedman's tests were performed, one for each group. Only the BF group showed significant results (*p* = 0.0011). On average, the BF group showed a higher amount of change between the three blocks compared with the BNF group together with a higher variability especially during the Middle and Late period (see Figure 7). At the end of the session and during the Last period (trials 16–20), the BF group showed, on average, 200% percent more than the BNF group on the scores of Sound instability together with a standard deviation 4.7 times higher., although This difference was not found to be significant (Wilcoxon rank-sum test: *p* = 0.2973).

### 3.2. EEG Analysis

The results of IGAE ranked beta and gamma band power at frontal sites as the most important features to discriminate between beginners and experts with a value of 0.01931 and 0.01905, respectively, followed by gamma band power at posterior sites with 0.00828; right frontal beta with 0.00811; right frontal gamma with 0.00562; left frontal gamma with 0.00424; left posterior gamma and beta with 0.00297 and 0.00269, respectively.

**Figure 8:**
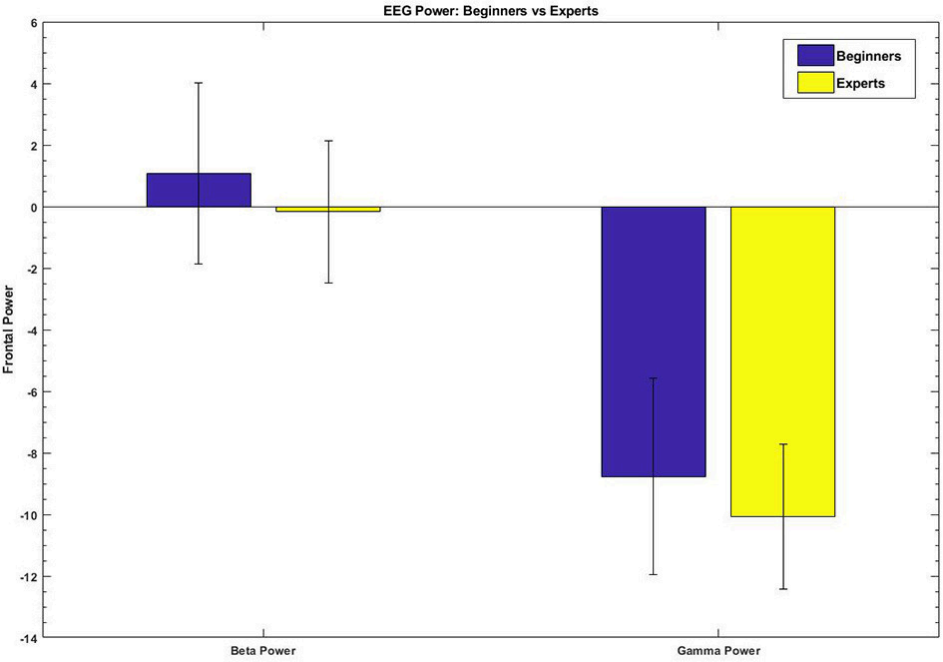
Beta and gamma band power measured at frontal sites during the first block (5 trials). Beginners are represented in blue and experts in yellow. Experts exhibited significant lesser values of frontal gamma band power when compared with beginners at both frequency bands.

A Shapiro-Wilk test for normality was performed in the distributions of each group showing significant results leading us again to use non-parametric statistical tests. Eight Wilcoxon rank-sum tests were performed for each feature comparing experts and beginners adjusting the *p*-value with Bonferroni correction. Only beta and gamma band power at frontal sites showed significant results (*p* < 0.00001 both). Figure 8 shows the differences in beta and gamma band power between beginners and experts. On average, Beginners showed an amount of 190% more of power than experts in the beta band and a 16% more in the gamma band.

**Figure 9:**
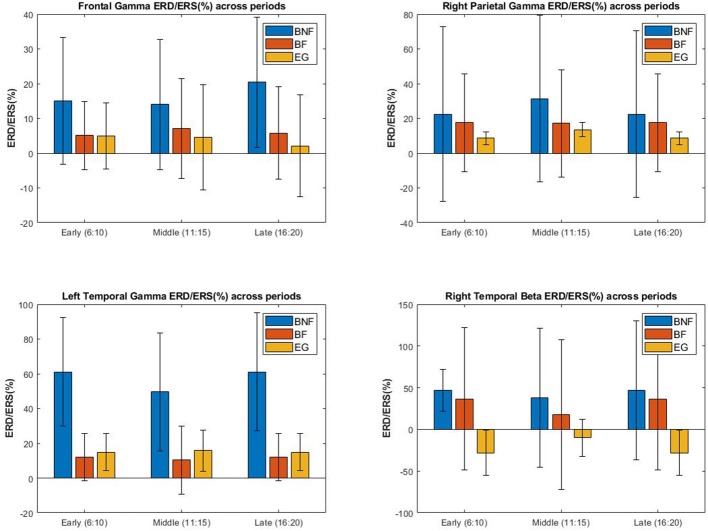
**Upper left Figure:** Frontal gamma ERD measured at each block using first five trials as baseline. Only the BNF and BF group showed a significant desynchronization through the experiment. Desynchronization of the BNF group was significantly higher than the one of the BF group. **Upper right Figure:** Here we can see the results for right parietal gamma ERD. Only the EG group showed a significant desynchronization through the experiment. **Bottom left Figure:** Left temporal gamma ERD. All the groups showed significant desynchronizations through the experiment. **Bottom right Figure:** Right temporal beta band ERD. Only the EG group showed a significant synchronization through the experiment.

ERD/ERS was computed for the rest of the blocks using the first block as a baseline. A Wilcoxon sign-rank test was performed for each cluster and frequency band to determine those sensor clusters and frequency bands where variations occurred through the rest of blocks. The *p*-value threshold chosen after the Bonferroni correction for 156 tests was *p* < 0.00032. Results showed an overall desynchronization of gamma band across the scalp for all the experimental groups and all the blocks with certain differences between them. Significant desynchronizations were obtained for gamma band at frontal electrodes in both groups of beginners (see Figure 9) but not for experts (*p* < 0.00032). Significant desynchronizations for gamma band were also found at right parietal and left temporal in all the groups including experts. Only the expert group showed significant changes at the right temporal cluster reflecting a synchronization of beta band.

Significant results were found when comparing the amount of desynchronization at frontal sites of the BNF group with the BF (*p* = 0.057) and with the EG(*p* = 0.025). Beta synchronization found at the right temporal cluster in experts showed significant results when comparing it with the BNF group (*p* = 0.000041) and with the BF group (*p* = 0.0149). In order to detect differences between the blocks, three more Friedman's tests were performed, one for each group. No significant changes were found in the amount of ERD/ERS between blocks.

### 3.3. Correlation Analysis

**Figure 10:**
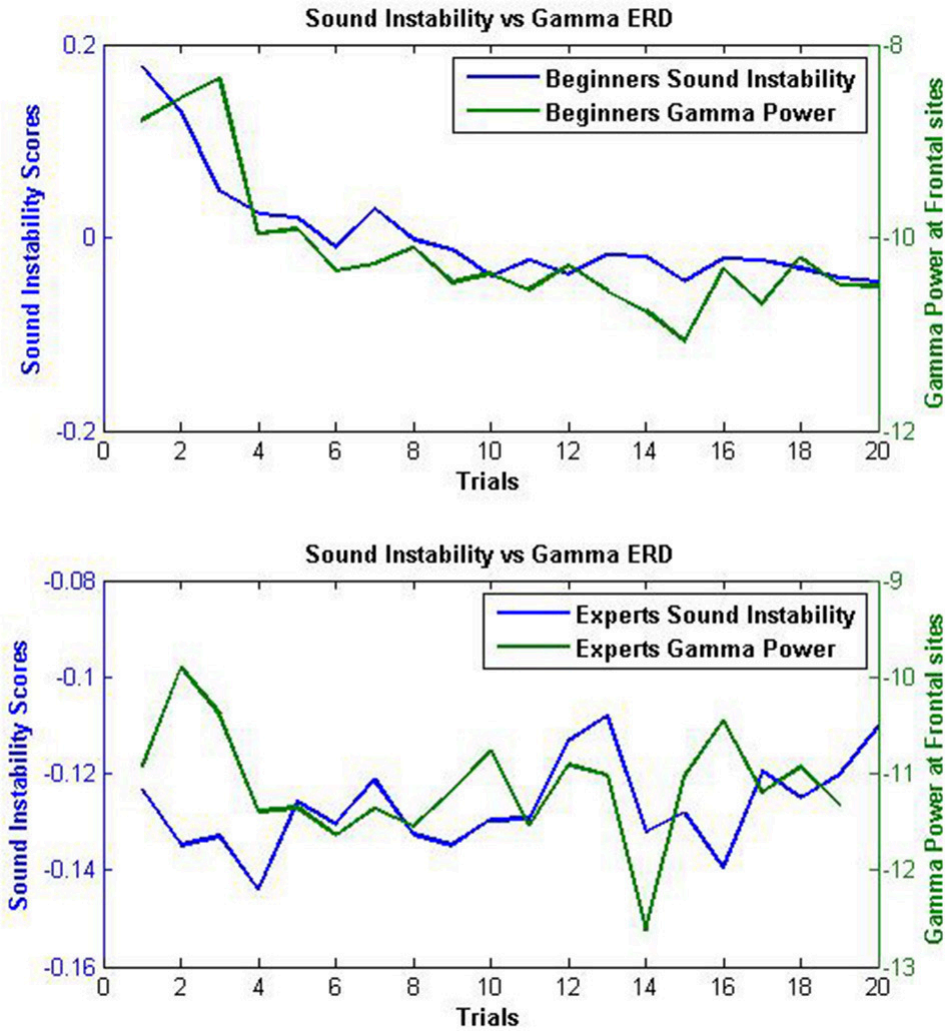
**Upper Figure:** Frontal gamma band power seen in comparison with the Sound instability scores of both beginners groups. **Bottom Figure:** Frontal gamma band power seen in comparison with the Sound instability scores of the expert group.

Four Pearson's correlations were performed in total. Four of them between each one of the four frequency bands filtered (theta, alpha, beta, gamma) from frontal electrodes and Sound instability across the whole session. After adjusting the *p*-value for four statistical tests results showed only one statistically significant linear correlation at gamma band (*R*^2^ = 0.70, *p* = 0.00001). In Figure 10 Sound instability results averaged for each trial for both beginners and expert groups can be seen in comparison with the averaged gamma band power at frontal electrodes obtained for each trial.

### 3.4. Behavioral Analysis

**Figure 11:**
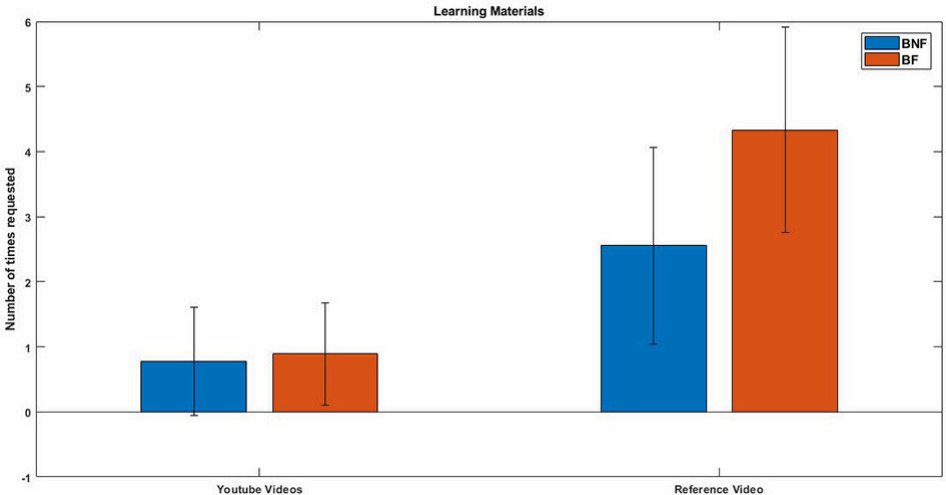
Number of times each group of beginners requested learning materials during the experiment.

The number of times each participant requested each learning material (instructional videos, reference video or their score evaluated with audio descriptors) was recorded and compared between the two different beginners groups (BF and BNF). A Shapiro-Wilk test for normality was performed in the distributions of the number of times each group requested a learning material showing significant results for the BNF group. Two statistical tests were performed in total adjusting the *p*-value with Bonferroni correction (*p* = 0.016). Two Mann-Whitney *U*-test were performed to find differences between both beginners group (BF + BNF) in each one of the distributions. No significant differences were found, although results show some different tendencies in terms of the number of reference video requests (*p* = 0.0203). On average, the BF group requested the reference video 25.8% more times than the BNF group. In Figure 11, we can see the differences found in each distribution for each group. The BF group also had the possibility to request the audio-based automatic evaluation of their performance produced by the system. A paired sampled *t*-test was performed between the number of times the BF group requested the reference video with the number of times they requested the audio evaluation. No significant differences were found (*p* = 0.37).

## 4. Discussion

In this work, we have used audio features like pitch stability and dynamic stability to measure sound quality as has been done in previously related work (Romaní et al., 2015; Giraldo et al., 2018). We have found that the aperiodicity measure is also a reliable indicator and offers extra information not found in the rest of descriptors. However, in this work we have not only shown how these descriptors could be useful to discriminate between those sounds performed in the violin by experts and beginners (i.e., a good or bad sound) but, in addition, we have used them to track the amount of learning of 18 participants, with no prior experience neither with the violin nor any bowed-string instrument, during 20 trials while learning to produce a stable and sustained sound in an open string. Allowing us to study objectively the impact of feedback technologies in the process of learning to produce a good sound with the violin.

The visual feedback considered in this study consisted of a sound quality indicator computed using audio descriptors extracted from the audio produced by participants. The feedback was presented offline to participants in the form of a graph where the sound quality of the last trial was shown relative to the previous ones. They could also compare their performance to that of an expert participant who previously did the experiment. We referred to this type of technology a sound quality visual feedback system (SQVFS).

At the end of the session, both groups of participants improved significantly their scores with the exception of the expert group. However, only participants who received feedback from the SQVFS improved their results between the different blocks in which the session was composed while the results of the rest of participants remained stable after the Early block. Nonetheless, no significant differences were found at the end of the session regarding the amount of improvement due to the fact that they also showed a higher variability during the last two blocks.

We hypothesize that the reason for the found variability during the last two blocks is that feedback encouraged participants to experiment with new ways of producing sound. After reaching a certain threshold of sound quality, the produced sound of participants starts to stabilize and requires experimentation in order to reach the quality of the one produced by an expert. Without the presence of feedback (like the one given by an SQVFS in this case) participants may find hard to detect by themselves how far their sound is from the one of the expert reference video. Experimenting new ways of displacing the bow would have been the only way for them to check if it led to an improvement in the results of the descriptors or not. Some participants would have improved their results while others may have tried different strategies without success reflecting thus, the greater variability seen in the results. This hypothesis is also supported by the results found in the behavioral analysis. Participants who received feedback from the SQVF requested, on average, a bigger number of times the reference video than the group without feedback, reflecting, in our opinion, bigger efforts to analyze the sound and technique from the video in order to replicate it in their own performance.

Regarding the electrophysiological analysis, we found beta and gamma band power at frontal sites to be the best features to discriminate between beginners and experts during the first block of the experiment. Beginners showed significantly higher activity of those bands than experts. No changes were found at the theta band as the neural efficiency hypothesis may have predicted (Klimesch et al., 1997; Bazanova and Aftanas, 2006) although changes found at beta and gamma bands are consistent with those found by Gutierrez and Ramírez-Moreno (2016) and with the temporal binding model (Bhattacharya et al., 2001; Howard et al., 2003; Fitzgibbon et al., 2004). Differences may be related to the way the EEG data was acquired and the type of task. In previous work (Salazar et al., 1990; Landers et al., 1994; Haufler et al., 2000), the effect of expertise in sports activity is measured using EEG signals during what is called the “pre-shot routine”, which is recorded just before the skilled movement occurs. Tasks like learning to type in a different keyboard layout as Gutierrez and Ramírez-Moreno (2016) studied or maintaining a stable sound with the violin during a period of time, require measuring the electrophysiological signals not before but, during the realization of the movement. The location of the electrodes in this experiment was also different due to hardware limitations of the Emotiv Epoc device with a more frontal density of electrodes and less density at parietal and central areas.

In fact, Gutierrez and Ramírez-Moreno (2016) found desynchronizations at both beta and gamma bands as participants started to learn the tasks. In our study, we also found desynchronizations across blocks in the gamma band at frontal sites that showed some degree of correlation with task improvement. The expert group, who did not show significant improvement along the session, neither exhibited significant desynchronizations at frontal gamma band as both groups of beginners did. This results may be interpreted from the temporal binding model which associates gamma band with the role of integrating (binding) information processed in distributed cortical areas. Task complexity demands more cognitive resources, more binding and thus, gamma band power enhancement, which may be reduced as the demanded task begins to be automated which could have been the case of both beginners groups.

We also found significant differences between the amount of gamma band desynchronization among beginners groups. On average, the BNF group showed higher desynchronizations than the BF group. This results may also be explained by our experimentation hypothesis. We hypothesize that the lower desynchronizations found at the BF group could reflect the higher efforts made during the task trying to achieve the expert score in the SQVF.

EG showed very clear localized gamma band desynchronizations at right parietal and left temporal sites and beta band synchronizations at right temporal area. Both groups of beginners also showed significant desynchronizations at the left temporal cluster, however, desynchronizations of the gamma band at right parietal and synchronizations at right temporal were exclusive from experts. This may indicate the use of different strategies when performing the task and maybe, a good reliable indicator to discriminate between beginners and experts. This could be exploited as a future neurofeedback protocol for violin students. Although the limited number of electrodes of the Emotiv Epoc prevents a deeper analysis of the results, its low cost and easy setup make it a good candidate to be used in educational environments or as a neurofeedback system.

In contrast with most part of the previous research that has studied the use of technology to provide different kinds of feedback to improve learning (Thorpe, 2002; Welch et al., 2004; Wilson et al., 2008; Van Der Linden et al., 2011; Wang et al., 2012; Leong and Cheng, 2014; Schlegel and Gregory Springer, 2018), we did not found statistical differences between the amount of improvement at the end of the task between the BF and the BNF group. However, behavioral differences found among the BF group could be the result of an increment of the conscious awareness of their own performance as suggested by Hamond (2017). It is important also to highlight that the demanded task and the feedback provided in this study differs widely compared with previous work which mainly studies pitch accuracy and uses real-time feedback. The amount of time could also have been insufficient considering that some previous research with novice violinists used up to six sessions (40 min. each) within a period of 8 days (Van Der Linden et al., 2011).

One limitation of the present study is the lack of qualitative analysis that could have been collected at the end of the experiment to explore the degree of effort and implication that participants deposited in the task and how much they valued the use of an SQVFS during their practice. However, questionnaires also have their limitations and behavioral results like the ones we showed may offer us the possibility to infer how much they valued the SQVF by considering the number of times they requested it.

This research could have benefited from optical motion capture techniques and gesture analysis as has been done before (Ng and Nesi, 2008; Schoonderwaldt and Demoucron, 2009; Deutsch, 2011). Tracking with more detail bow movements of participants along the session would have allowed us to study with more detail the amount and type of bow movement experimentation. A higher number of electrodes in the EEG would have also been beneficial since it would have allowed us to study changes at central sensorimotor areas and thus, to see clearly if the results still coincide with those found by Gutierrez and Ramírez-Moreno (2016).

As motivation seems to be an important variable to take into account when evaluating learning processes (Elwell and Grindley, 1938), future work could address optimal ways to measure it. Different experimental designs may be proposed to test motivation, for example allowing participants to do as many trials as they want and to stop whenever they want. If a significant difference on the time spent learning is found between groups (technology and no-technology groups), this would indicate that the motivation offered by this kind of tools may be considered to be an important factor on the learning process. EEG data collected from participants could also be a reliable indicator to measure differences among participants and how cognitive load or boredom may influence their decision to stop the task and leave.

Finally, another important limitation of the study is the small number of participants involved, although results obtained seem promising. It is also important to take into account that both beginner groups consisted of musicians due to their greater accessibility on the campus. Although we hypothesize that this type of task could have been performed similarly by both non-musicians and musicians (given that beginner participants had no experience in violin or related instruments), it is known that musicians show different patterns of brain activation than non-musicians in a wide variety of tasks (Bhattacharya et al., 2001; Sokolov et al., 2004; Gurtubay et al., 2006; Shahin et al., 2008; Trainor et al., 2009). This means that we may found different electrophysiological results with non-musicians than those founds with musicians. Future work is needed to investigate if the results of this study replicate in different contexts.

## 5. Conclusions

In this work, we have studied the effects of an SQVFS in violin beginner students while learning to produce a stable sound using the bow. A group of experts was included in the study as reference. Experts did not show improvement along the session, while both groups of beginners did. In particular, only the BF group (beginners with SQVF) showed improvement through the Middle and Late blocks of the session while the BNF group (beginners without SQVF) stabilized their results after the Early block. We hypothesize that SQVF increased the awareness of participants about how far they were from an expert performance, leading them to experiment more with the instrument and getting more involved in the task. The BF group also requested the reference video more times compared with BNF.

Higher values of gamma and beta band power were found at frontal sites of both BNF and BF group when compared with EG during the first block. However, only beginners showed significant gamma band desynchronizations across blocks that showed some correlation with the amount of improvement in the task. This leads us to propose gamma band as a potential biomarker of motor learning similarly to Gutierrez and Ramírez-Moreno (2016). Task complexity demands more cognitive resources, more binding and thus, gamma band power enhancement, which may be reduced as the demanded task begins to be automated as could be the case found of both beginners groups. Nonetheless, the BNF group showed a higher amount of desynchronization than the BF group. This results could also be interpreted from our experimentation hypothesis as lower desynchronizations found at the BF group could reflect higher efforts made during the task trying to achieve the expert score.

## Data Availability Statement

The datasets generated for this study can be found in https://doi.org/10.5281/zenodo.2072946 and https://github.com/adavidBlancoUPF/Evaluation-of-Audio-Based-Feedback-Technologies/.

## Author Contributions

AB and RR designed the methodology of the study. AB recorded, processed and analyzed the EEG and audio data, and wrote the paper. RR supervised the study and contributed to the writing of the paper.

### Conflict of Interest Statement

The authors declare that the research was conducted in the absence of any commercial or financial relationships that could be construed as a potential conflict of interest.
